# Our Philadelphia Letter

**Published:** 1886-10

**Authors:** Harry Huzza

**Affiliations:** Philadelphia, Pa.


### Correspondence.

### OUR PHILADELPHIA LETTER.

THE OPENING OF THE MEDICAL COLLEGES—JEFFERSON'S NEW PROFESSOR OF ANATOMY—OTHER CHANGES IN JEFFERSON—CLINICAL NOTES, ETC.

*Editors Atlanta Medical and Surgical Journal:*

There is always a quiet serenity at the medical colleges before the fall and winter session begins—a kind of calm it is that precedes the storm—a simple waiting for what is to come. Soon the students straggle in by twos and threes, and before one realizes it, the rush comes, the seats are filled, and without jar or confusion, the first clinic or the opening lecture greets the crowded benches. There is something interesting to a philosopher in the "Presto, change! Now you don't see them, and now you do!" This bids fair to prove a good year for all the colleges. While there have been disasters in many places, yet prosperity seems to smile upon the country; business is opening briskly and the farmers' good crops give impetus to all avocations of life.

At Jefferson the students are matriculating early and in large numbers. Things look quiet, but behind the scenes all preparations have and are being made for a thorough winter's work. The resignation of Professor Wm. H. Pancoast left vacant the chair of anatomy. This will be efficiently filled by Dr. W. S. Forbes. Dr. Forbes will deliver the introductory lecture of the fall course on the evening of September 30th, 1886. He is a thorough anatomist and a careful teacher, so the instruction in this line at Jefferson will be good. Some considerable changes have been made in different branches that will extend the facilities for useful work. There will now be three demonstrators and three assistant demonstrators of anatomy, thus insuring supervision and instruction of all the students. Moreover, the laboratories will be distributed over the first as well as the second and third year's work. Jefferson continues to follow the progressive movement for raising the standard of medical education, which is so much needed.

I saw Emmet's operation on the perineum performed for rectocele, and, by the latest improvements, it is certainly rendered the best. It is very simple, but, like most simple things, very difficult to explain. Cat-gut sutures, prepared by Lister's latest formula, were used instead of silver wire. The results in four cases operated during the last two weeks have been most excellent. A case of inverted uterus may be worth noting because not common. The woman had a child five years old, but the history of the invertion was indefinite. Continued hemorrhage threatened life. It was reduced by steady, persistent pressure with the fingers on the cervical portion, alternating with White's uterine repositor on the fundus. Ether was of course used and two hours' work required to accomplish the restoration. One of the out-patients demonstrated the absolute inocuousness of silver wire in the tissues. She had been operated for lacerated perineum four years ago, and, by an oversight, one of the shotted silver-wire sutures had been allowed to remain. It gave no trouble at all and was not noticed till she lately married, when the shot proved inconvenient to her husband.

There has been as yet very little clinical work. Nothing "new" nor startling seems to agitate the Æsculapian branch of science. Will try to chronicle something of the kind next month. Yours truly,

HARRY HUZZA.

*Philadelphia, Pa., Sept. 20th, 1886.*

